# Nemabiome metabarcoding reveals strongyle parasite diversity in wild deer in Victoria, Australia

**DOI:** 10.1007/s00436-026-08722-1

**Published:** 2026-07-08

**Authors:** Jacqui Panozzo, Dhanasekaran Sakthivel, Robin B. Gasser, Wendy Wright, Grant Rawlin, David Piedrafita

**Affiliations:** 1 Institute of Innovation, Science and Sustainability, Federation University, Gippsland, VIC 3806 Australia; 2 Department of Biomaterials, Saveetha Dental College and Hospital (SDCH), Saveetha Institute of Medical and Technical Sciences, Saveetha University, Chennai, 600077 India; 3 Department of Veterinary Biosciences, Faculty of Science, Melbourne Veterinary School, The University of Melbourne, Parkville, VIC 3010 Australia; 4 Department of Jobs Precincts and Regions, Agriculture Victoria Research, Centre for AgriBioscience, La Trobe University, Bundoora, VIC 3086 Australia

**Keywords:** Nemabiome metabarcoding, Gastrointestinal nematodes, Wild deer, Strongyle parasites, ITS-2, Wildlife–livestock interface

## Abstract

Interactions between wild deer and domestic livestock are increasingly common in southeastern Australia, but contemporary information on gastrointestinal nematodes infecting wild deer remains limited. In this study, nemabiome metabarcoding of the internal transcribed spacer 2 (ITS-2) region of ribosomal DNA was used to characterise strongyle communities in wild deer from Victoria, Australia. A total of 194 faecal samples from three deer species – sambar (*Rusa unicolor*), fallow (*Dama dama*) and hog deer (*Axis porcinus*) – were analysed using Illumina amplicon sequencing. Strongyle DNA was detected in 104 samples, revealing eleven taxa. Eight species (*Cooperia oncophora*, *Haemonchus* spp., *Ostertagia ostertagi*, *O. leptospicularis*, *O. radiatum*, *Trichostrongylus axei*, *T. colubriformis* and *T. vitrinus*) are commonly associated with domestic ruminants, whereas the cervid-associated strongyle *Spiculopteragia asymmetrica* was the most frequently detected taxon. Deer sampled near farmland harboured significantly greater strongyle diversity than those from more remote locations. Overlapping parasite taxa were also detected in co-grazing deer and cattle. These findings indicate that wild deer in southeastern Australia harbour diverse strongyle communities and highlight the potential for overlapping parasite assemblages at the wildlife–livestock interface.

**Supplementary Information:**

The online version contains supplementary material available at https://doi.org/10.1007/s00436-026-08722-1.

## Introduction

Deer are not native to Australia and were introduced during the early nineteenth century primarily for hunting and game management (Moriarty 2004). Today, free-ranging populations are established across multiple regions of the country. In the southeastern state of Victoria, four deer species are widely distributed and have well-established populations: the Asiatic sambar (*Rusa unicolor*), hog deer (*Axis porcinus*), fallow deer (*Dama dama*) and red deer (*Cervus elaphus*). Although further introductions have ceased, deer populations in Victoria continue to expand in both abundance and geographic range, facilitated by extensive forested landscapes and the historical classification of deer as protected game species (Victoria’s State of the Forests Report 2013, Moloney and Turnbull 2014; Gray 2016). Estimates suggest that wild deer populations in Victoria now exceed one million animals (McLennan 2016; Ray and Burgman 2006).

The increasing abundance and distribution of wild deer raise significant ecological and agricultural concerns, particularly where deer occupy landscapes shared with domestic livestock (Sommerville and Condon 2018). As ruminants, deer share similar digestive physiology with domesticated cattle, sheep and goats, making them susceptible to many of the same gastrointestinal (GI) parasites. Gastrointestinal nematode infections, particularly with strongyle species, are ubiquitous in pasture-based livestock production systems and remain a major constraint on animal productivity worldwide, causing reduced weight gain, lower milk production, and substantial economic losses (Durie 1961; Morgan et al. 2004; Charlier et al. 2014). While extensive research in Europe and North America has demonstrated that wild deer commonly host economically-important strongylid genera such as *Ostertagia*, *Haemonchus*, and *Trichostrongylus* (Al Saqur et al. 1980; Sleeman 1981; Biset et al. 1984; Barth and Matzke 1984; Ambrosi et al. 1993; Cisek et al. 2003; Vengušt and Bidovec 2003; Balicka-Ramisz et al. 2005; Irvine et al. 2006; Böhm et al. 2007; Cerutti et al. 2010; Omeragić and Zuko 2011; Rehbein et al. 2014; Beaumelle et al. 2021), Australian data remains critically outdated. The last published investigation of GI nematodes in Australian wild deer was conducted more than three decades ago (McKenzie et al. 1985). Given the massive expansion of deer populations since that time, investigating their current role in parasite ecology at the wildlife-livestock interface is highly warranted.

Characterising gastrointestinal parasite communities presents several cross methodological challenges in both livestock and wildlife research. Conventional diagnostic techniques, such as faecal egg counts (FEC), provide poor taxonomic resolution because the eggs of most strongylid nematodes are morphologically indistinguishable. While larval culture can improve identification, it is frequently biased by differential larval development and recovery rates. To bypass these limitations, wildlife parasitology has historically relied on necropsy-based examinations of the host gastrointestinal tract (Batty et al. 1987; Pybus 1990; Vengušt and Bidovec 2003; Shimalov and Shimalov 2003; Irvine et al. 2006; Santín-Durán et al. 2008; Bolukbas et al. 2012; Flueck and Smith-Flueck 2012; Rehbein et al. 2014). While highly informative, necropsies are logistically demanding, necessitate small sample sizes, and fail to offer a scalable strategy for ongoing, non-invasive surveillance.

Recent advances in molecular diagnostics offer a powerful alternative to overcome these diagnostic limitations. Next-generation sequencing (NGS) approaches have been successfully applied to analyze strongylid nematode communities via metabarcoding of the internal transcribed spacer 2 (ITS-2) region of ribosomal DNA (rDNA). This approach, termed “nemabiome” metabarcoding, allows for the simultaneous detection and species-level identification of multiple nematode species from mixed parasite communities within faecal samples (Avramenko et al. 2015, 2017). The ITS-2 region serves as an ideal molecular marker due to its low intraspecific variation and distinct interspecific sequence differences (Gasser 2006, 2025; Gasser et al. 2008). Amplification using the broadly conserved primers NC1 and NC2 enables high-throughput, comprehensive profiling of strongylid diversity without the biases of traditional culture or the constraints of post-mortem examinations (Gasser et al. 1993; Beaumelle et al. 2021).

The present study applied nemabiome metabarcoding of the ITS-2 rDNA region to characterise the gastrointestinal strongyle communities of wild deer in southeastern Victoria, Australia. Faecal samples were collected from three co-occurring deer species, sambar, fallow and hog deer, to assess the baseline diversity of strongylid nematodes present in wild populations. In addition, samples from sympatric cattle grazing on properties where wild deer were also present were analysed to explore the co-occurrence of strongyle species across wildlife and livestock.

## Materials and methods

### Sample collection

Faecal samples from wild deer were collected between 2015 and 2017 at multiple sites across Victoria, Australia. A total of 194 samples were obtained from sambar (*Rusa unicolor*, *n* = 80), fallow (*Dama dama*, *n* = 44) and hog deer (*Axis porcinus*, *n* = 70). Sambar samples were collected primarily from hunted animals in eastern Victorian forests. Fallow samples were collected from bushland sites across eastern Victoria, and hog deer samples were obtained during necropsies conducted during Parks Victoria culling operations at Wilson’s Promontory National Park. Ground-collected samples were assigned to species based on regional distributions and scat morphology (Triggs 1996). Hog deer culling formed part of a pest control program. Sambar deer samples were collected throughout the hunting season (winter 2016), hog samples were collected in December (2015, 2017).

Faecal samples of co-grazing deer and cattle were also collected on three properties in southeastern Victoria: Toora (38.6152°S, 146.3074°E), Tonimbuk (38.0127°S, 145.7144°E) and Neerim East (37.9350°S, 146.0096°E). In total, 61 deer samples and 35 cattle samples were collected across the three properties around March of 2017.

### Classification of sampling sites relative to farmland

Sampling locations were classified by proximity to farmland: ≤5 km (“near farmland”) or > 5 km (“remote”). This threshold was based on published home-range estimates for fallow, hog, sambar and rusa deer, which suggests that deer have home ranges of less than 5 km in width (Borkowski and Pudełko 2007; Spaggiari and Garine-Wichatitsky 2006; Odden and Wegge 2007; Kamil et al. 2001; Morse et al. 2009).

### Faecal egg counts (FECs) and strongyle egg isolation

For FECs, 5 g of faeces was homogenised in 50 ml saturated sodium nitrate solution (specific gravity 1.3 g ml⁻¹), filtered through 250 μm and 100 μm sieves and adjusted to 50 ml. A 1 ml aliquot was used to determine eggs per gram (EPG) using a modified McMaster method using a Whitlock counting chamber (sensitivity 10 EPG). The remaining suspension was centrifuged at 1500 × g for 5 min. The supernatant was passed through a 25 μm sieve to retain eggs, rinsed with tap water and centrifuged at 2000 × g for 10 min to pellet material. Isolated eggs were stored at − 20 °C until DNA extraction.

### DNA extraction and PCR-based sequencing

Genomic DNA was extracted from isolated eggs using the DNeasy PowerSoil Kit (Qiagen, Australia) according to the manufacturer’s instructions. Samples were screened for strongyle DNA using primers NC1 and NC2 targeting the internal transcribed spacer 2 (ITS-2) region of nuclear ribosomal DNA (Supplementary Table 1) (Gasser et al. 1993). Amplicons (~ 300–350 bp) were visualised on 2% agarose gels (Supplementary Fig. 1).

Individual deer samples that were positive for strongyle DNA via NC1/NC2 primers, were then analysed via amplicon next-generation sequencing. For the samples collected on farms, strongyle DNA-positive samples were pooled based on species (deer or cattle) and study site. For each pool, equal volumes of individual samples was used to make a total of 30uL for analysis. Positive samples were re-amplified with adaptor-tagged primers following established protocols, PCR cycle conditions and molecular reagents by Avramenko et al. 2015. Amplicons (~ 400 bp) were verified by electrophoresis and sequenced on an Illumina MiSeq platform at the Australian Centre for Ecogenomics. Negative controls (no-template reactions) were included during PCR amplification and sequencing to monitor potential contamination, and no amplification was detected in these controls.

### Sequence analysis and taxonomic assignment

Sequence processing was performed using the open source online software Galaxy (Afgan et al. 2018). Paired-end reads were merged and converted to FASTA format (Blankenberg et al. 2010). Sequences were aligned against a curated ITS-2 reference database derived from morphologically identified strongylid nematodes (Supplementary Table 2) using MegaBLAST (Camacho et al. 2009; Cock et al. 2015). The custom database was based off that used by Avramenko et al. (2015), with the addition of deer specific strongyle nematodes such as *Spiculopteragia asymmetrica* and possible environmental contaminates, such a soil nematode *Heterodera spp*. and marsupial strongyles *Macropostrongyloides spp*.

Following alignment any sequences with an alignment of < 200 bp were excluded. Taxonomic assignment used a 99% similarity threshold, except for *Cooperia oncophora* (96%), *Trichostrongylus vitrinus* (97%), *Haemonchus* spp. (95%) and *Ostertagia leptospicularis* (95%).

*Haemonchus* species identification was left at a genus level, due to possible confusion about taxonomy of *H. placei* and *H. contortus*, Reads representing < 0.05% of total reads per sample were considered artefacts and removed. Across all samples, sequencing generated approximately 9,434,698 reads after quality filtering, with a mean of approximately 85,770 reads per sample. Detailed sequencing statistics are provided in Supplementary Table 3.

## Results

A total of 194 faecal samples from wild deer were analysed using ITS-2 amplicon sequencing to characterise strongyle communities in three host species: sambar (*Rusa unicolor*, *n* = 80), fallow (*Dama dama*, *n* = 44) and hog deer (*Axis porcinus*, *n* = 70). Initial screening by faecal egg counts detected strongyle eggs in 19 samples (15.1%), with a mean intensity of 45 eggs per gram (EPG) (range 10–200 EPG). PCR amplification of ITS-2 sequences detected strongyle DNA in 117 samples (60.3%). After removal of sequences corresponding to non-ruminant nematodes (e.g. *Heterodora* spp, *Cloacina* spp, *Macropostrongyloides* spp) and background amplification, 104 samples (53.6%) were retained for nemabiome analysis.

High-throughput sequencing identified DNA from eleven strongyle taxa (Table 1): *Cooperia oncophora*, *Haemonchus* spp., *Oesophagostomum venulosum*, *O. radiatum*, *Ostertagia leptospicularis*, *O. ostertagi*, *Spiculopteragia asymmetrica*, *S. spiculoptera*, *Trichostrongylus axei*, *T. colubriformis* and *T. vitrinus*. Eight of these taxa (*C. oncophora*, *Haemonchus* spp., *O. ostertagi*, *O. leptospicularis*, *O. radiatum*, *T. axei*, *T. colubriformis* and *T. vitrinus*) are commonly associated with domestic ruminants, and DNA from one or more of these species was detected in 45 samples (23.3%). The cervid-associated genus *Spiculopteragia* was the most frequently detected group. DNA from *Spiculopteragia asymmetrica* occurred in 42.8% of samples and represented 79% of strongyle-positive samples; it was detected in all strongyle-positive samples from hog deer and in 78.4% and 48.3% of strongyle-positive fallow and sambar samples, respectively. The second most frequently detected species was *Oesophagostomum venulosum* (23.7%), which occurred in all three deer species. Detection frequencies differed among host species, with strongyle DNA detected most frequently in fallow deer (84.1% of samples), followed by hog deer (55.7%) and sambar deer (36.3%).

**Table 1:** Strongyle taxa detected in individually tested deer faecal samples using ITS-2 amplicon sequencing. Strongyle DNA in faecal samples via amplicon NGS of ITS-2 regions was identified using a reference database (**Supplementary Table 2**). Data are presented as number of positive samples, and percentage of faecal samples in which DNA of each strongyle taxon was detected. ND = not detected

		Economically important strongyle species								Other strongyle species		
		*C. oncophora*	*Haemonchus* spp.	*O. leptospicularis*	*O. ostertagi*	*O. radiatum*	*T. axei*	*T. colubriformis*	*T. vitrinus*	*S. asymmetrica*	*S. spiculoptera*	*O. venulosum*
Total (n = 194)	n +ve	5	9	13	15	4	6	1	5	82	7	46
	%	2.6	4.6	6.7	7.7	2.1	3.1	0.5	2.6	42.8	3.6	23.7
Sambar (n = 80)	n +ve	5	4	4	14	3	6	ND	2	14	6	17
	%	6.3	5.0	5.0	17.5	3.8	7.5		2.5	17.5	7.5	21.3
Fallow (n = 44)	n +ve	ND	5	9	ND	ND	ND	1	ND	29	ND	28
	%		11.4	20.5				2.3		65.9		63.6
Hog (n = 70)	n +ve	ND	ND	ND	1	1	ND	ND	3	39	1	1
	%				1.4	1.4			4.3	55.8	1.4	1.4

**Fig. 1:**
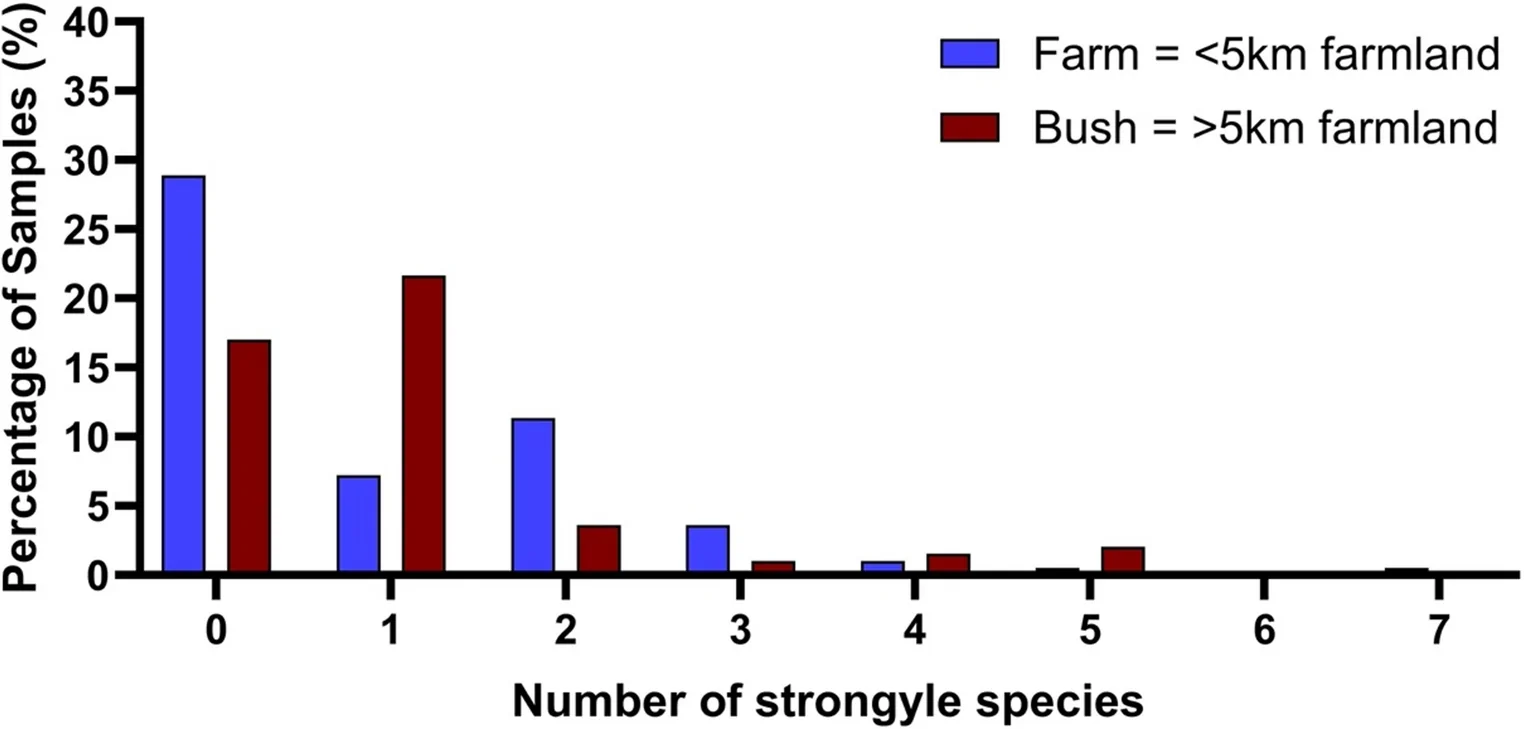
Distribution of the number of strongyle taxa detected in faecal samples from wild deer in Victoria, Australia, according to proximity to farmland. Strongyle taxa were identified by next-generation sequencing (NGS) of ITS-2 amplicons. Bars show the percentage of samples containing the indicated number of strongyle taxa for samples collected ≤ 5 km from farmland (“Farm”) and ≥ 5 km from farmland (“Bush”). Data represent 194 deer faecal samples

**Fig. 2:**
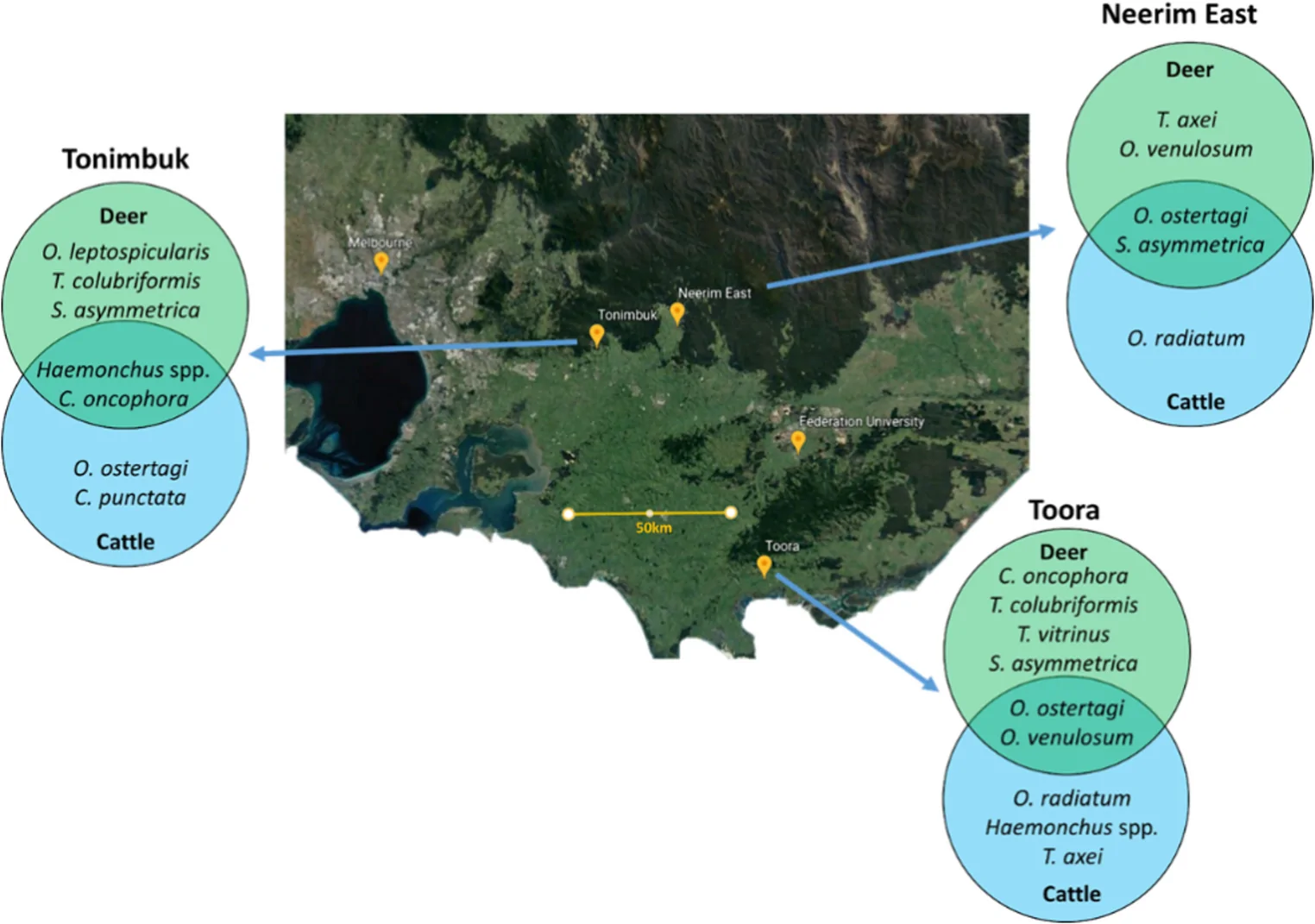
Locations of three cattle farming properties in southeastern Victoria, Australia (Tonimbuk, Neerim East and Toora) where faecal samples from co-grazing cattle and deer were collected. Strongyle taxa detected by next-generation sequencing (NGS) of pooled ITS-2 amplicons are shown for deer (upper portion of each circle) and cattle (lower portion)

When samples were classified according to proximity to farmland, deer collected within 5 km of agricultural land showed a higher frequency of infections with multiple strongyle taxa. Multiple species (≥ 2 taxa) were detected in 70.2% of samples collected near farmland compared with 27.6% of samples collected more than 5 km from farmland (Fisher’s exact test, *p* < 0.001) (Fig. 1). Analysis of pooled samples from deer and cattle grazing on the same properties revealed several overlapping strongyle taxa (Fig. 2). *Ostertagia ostertagi* was detected in both host species at Toora and Neerim East. Additional taxa present in both deer and cattle included *Haemonchus* spp. and *Cooperia oncophora* at Tonimbuk, *Oesophagostomum venulosum* at Toora and *Spiculopteragia asymmetrica* at Neerim East. Other taxa were detected only in one host species at a given location.

## Discussion

Using ITS-2 nemabiome metabarcoding, this study characterised gastrointestinal strongyle communities in wild deer in southeastern Australia. Sequencing of ITS-2 amplicons from faecal samples of three deer species revealed eleven strongyle taxa, including several parasites of critical importance to domestic ruminant production systems. These findings indicate that wild deer in Victoria harbour diverse strongylid assemblages and that parasite communities in wildlife and livestock overlap significantly where these hosts share grazing environments. Among the taxa detected were species commonly associated with livestock, including *Cooperia oncophora*, *Haemonchus* spp., *Ostertagia ostertagi*, *O. leptospicularis*, *Trichostrongylus axei*, *T. colubriformis*, and *T. vitrinus*. Comparable observations have been reported from cervid populations in Europe and North America (Vengušt and Bidovec 2003; Irvine et al. 2006; Rehbein et al. 2014; Beaumelle et al. 2021). However, within the specific context of Australian grazing systems—where deer populations have expanded unchecked for over three decades without updated parasitological surveillance—these findings provide crucial evidence that wild deer may act as environmental multipliers or biosecurity reservoirs for production-limiting livestock parasites.

The dominant strongyle detected in this study was *Spiculopteragia asymmetrica*, a species widely recognised as a characteristic parasite of cervids. Its high frequency across samples suggests that it represents a core, self-sustaining component of the gastrointestinal strongyle community in deer populations in Victoria. In contrast, parasites more typically associated with cattle, such as *Ostertagia ostertagi* and *Cooperia oncophora*, were detected less frequently. This community structure demonstrates that while Victorian deer maintain their own host-adapted strongyle fauna, they actively acquire and harbor a subset of species from domestic livestock. Notably, we observed a greater diversity of strongyle species in deer sampled near farmland compared with those from remote forested locations. This gradient strongly indicates that proximity to livestock grazing systems alters wildlife parasite profiles. Fragmented agricultural fringes in southeastern Australia create high-density contact zones where deer encounter a broader range of infective larval stages deposited by livestock on improved pastures, accelerating cross-species parasite circulation (Morgan et al. 2004).

Analysis of pooled samples from deer and cattle on co-grazed properties confirmed that *O. ostertagi*, *Haemonchus* spp., and *C. oncophora* were concurrently present in both host types at the same geographic locations. While these spatial overlaps demonstrate that deer and cattle share a parasite fauna in communal environments, molecular detection of faecal DNA does not confirm active, patent cross-infection or define the direction of spillover. Consequently, while our results expose a porous wildlife–livestock interface in Victorian agricultural landscapes, distinguishing whether deer function as true reservoir hosts or merely incidental, dead-end hosts remains an essential epidemiological priority.

A notable finding in this study was the marked discrepancy between standard faecal egg counts (FEC) and molecular detection rates. Out of 185 total samples, 98 yielded negative results via conventional FEC but were subsequently confirmed positive via egg isolation and successful NC1/NC2 PCR amplification. Traditional flotation-based FEC methods are well-documented to have limited diagnostic sensitivity, frequently returning false negatives in low-intensity or subclinical infections common to free-ranging wildlife (Cringoli et al. 2004, 2010; Rinaldi et al. 2011; Mes et al. 2001). The high proportion of FEC-negative but PCR-positive samples demonstrates that the sequential egg isolation and molecular protocols employed in this study offer vastly superior diagnostic sensitivity compared to traditional microscopy (Mes et al. 2001, 2007). Relying solely on conventional FEC would have substantially underestimated the true prevalence of strongyle infections across the surveyed deer populations. These results underscore the necessity of integrating sensitive molecular tools into wildlife surveillance frameworks to accurately map parasite distribution and avoid false-negative biases at the wildlife–livestock interface.

A key limitation of utilizing fecal-derived ITS-2 metabarcoding data is that read counts cannot be reliably used to infer true adult worm burdens, infection intensity, or parasite viability due to variations in species-specific egg shedding rates and PCR amplification biases. Furthermore, while the ITS-2 marker excels at species-level discrimination, its highly conserved nature lacks the micro-evolutionary resolution required for population-level transmission analysis (Gasser 2006). To definitively resolve whether the parasite populations circulating in deer and livestock are genetically identical and actively interchanging, future investigations must deploy more polymorphic genetic markers. Specifically, the application of high-throughput mitogenomics-utilising next-generation sequencing of complete or near-complete mitochondrial genomes—offers the necessary haplotypic resolution to track fine-scale transmission dynamics and gene flow between wildlife and livestock populations (McLean et al. 2023; Gasser 2025). Integrating these advanced multi-locus molecular techniques with targeted necropsy-based worm counts will be critical to fully unravelling the epidemiology of intersecting parasite communities at this expanding interface.

In conclusion, wild deer in southeastern Australia harbour diverse communities of gastrointestinal strongylid nematodes, including species of high economic significance to the livestock industry. The occurrence of overlapping parasite taxa in deer and cattle in shared grazing environments indicates that parasite communities intersect dynamically at the wildlife–livestock interface. This baseline study underscores the need for modernized, multi-species biosecurity strategies that account for the role of free-ranging wildlife in parasite persistence across Australian agricultural landscapes.

## Supplementary Information

Below is the link to the electronic supplementary material.

ESM 1 (DOCX 970 KB)

ESM 2 (DOCX 33.8 KB)

## Data Availability

The data supporting the conclusions of this article are included within the article and as supplementary materials. The raw FASTQ sequencing data generated during this study have been deposited in the Short Read Archive (SRA) at the National Center for Biotechnology Information (NCBI) under the BioProject accession number PRJNA1472849.

## References

[CR1] Afgan E, Baker D, Batut B, van den Beek M, Bouvier D, Cech M, Chilton J et al (2018) The Galaxy platform for accessible, reproducible and collaborative biomedical analyses: 2018 update. Nucleic Acids Res 46:W537–W544 10.1093/nar/gky379 PMC6030816 29790989

[CR2] Al Saqur I, Armour J, Bairden K, Dunn AM, Jennings FW (1980) *Ostertagia leptospicularis* Asadov 1953 as a pathogen in British cattle. Vet Rec 107:511–513 10.1136/vr.107.22.511 7445359

[CR3] Ambrosi M, Manfredi MT, Lanfranchi P (1993) Pattern of abomasal helminths in fallow deer farming in Umbria. Vet Parasitol 47:81–86 10.1016/0304-4017(93)90178-p 8493770

[CR4] Avramenko RW, Redman EM, Lewis R, Yazwinski TA, Wasmuth JD, Gilleard JS (2015) Exploring the gastrointestinal nemabiome: deep amplicon sequencing to quantify species composition of parasitic nematode communities. PLoS ONE 10:e0143559 10.1371/journal.pone.0143559 PMC4668017 26630572

[CR5] Avramenko RW, Redman EM, Lewis R, Bichuette M, Palmeira BM, Yazwinski TA, Gilleard JS (2017) The use of nemabiome metabarcoding to explore gastrointestinal nematode species diversity and anthelmintic treatment effectiveness in beef calves. Int J Parasitol 47:893–902 10.1016/j.ijpara.2017.06.006 28797791

[CR6] Balicka-Ramisz A, Pilarczyk B, Ramisz A, Cisek A (2005) Occurrence of gastrointestinal and pulmonary nematodes of fallow deer in north-west Poland. Acta Parasitol 50:94–96

[CR7] Barth D, Matzke P (1984) Gastro-intestinal nematodes of fallow deer (*Dama dama* L.) in Germany. Vet Parasitol 16:173–176 10.1016/0304-4017(84)90018-9 6543050

[CR8] Batty AF, Chapman DI, Chapman N (1987) Prevalence of nematode parasites in wild fallow deer (*Dama dama*). Vet Rec 120:599 10.1136/vr.120.25.599-a 3629863

[CR9] Beaumelle C, Redman EM, de Rijke J et al (2021) Metabarcoding in two isolated populations of wild roe deer (*Capreolus capreolus*) reveals variation in gastrointestinal nematode community composition between regions and among age classes. Parasit Vectors 14:587 10.1186/s13071-021-05087-5 PMC8642965 34863264

[CR10] Biset S, Kleinjan E, Vlassoff A (1984) Development of Ostertagia leptospicularis in cattle. Vet Parasitol 16:23–33 10.1016/0304-4017(84)90005-0 6543051

[CR11] Blankenberg D, Gordon A, Von Kuster G, Coraor N, Taylor J, Nekrutenko A (2010) Manipulation of FASTQ data with Galaxy. Bioinformatics 26:1783–1785 10.1093/bioinformatics/btq281 PMC2894519 20562416

[CR12] Böhm M, White PCL, Chambers J, Smith L, Hutchings MR (2007) Wild deer as a source of infection for livestock and humans in the UK. Vet J 174:260–276 10.1016/j.tvjl.2006.11.003 17258479

[CR13] Bolukbas CS, Gurler AT, Beyhan YE, Acici M, Umur S (2012) Helminths of roe deer (Capreolus capreolus) in the Middle Black Sea Region of Turkey. Parasitol int 61(4):729–730 10.1016/j.parint.2012.06.008 22766455

[CR15] Borkowski J, Pudełko M (2007) Forest habitat use and home-range size in radio-collared fallow deer. Ann Zool Fenn 44:107–122

[CR16] Camacho C, Coulouris G, Avagyan V, Ma N, Papadopoulos J, Bealer K, Madden TL (2009) BLAST+: architecture and applications. BMC Bioinformatics 10:421 10.1186/1471-2105-10-421 PMC2803857 20003500

[CR17] Cerutti MC, Citterio CV, Bazzocchi C, Epis S, D’Amelio S, Ferrari N, Lanfranchi P (2010) Genetic variability of *Haemonchus contortus* in alpine ruminants. J Helminthol 84:276–283 10.1017/S0022149X09990587 19889245

[CR18] Charlier J, van der Voort M, Kenyon F, Skuce P, Vercruysse J (2014) Chasing helminths and their economic impact on farmed ruminants. Trends Parasitol 30:361–367 10.1016/j.pt.2014.04.009 24888669

[CR19] Cisek A, Balicka-Ramisz A, Ramisz A, Pilarczyk B (2003) Occurrence of gastrointestinal nematodes in cervids (Cervidae) of north-west Poland. Electron J Pol Agric Univ 6

[CR20] Cock PJA, Chilton JM, Grüning B, Johnson JE, Soranzo N (2015) NCBI BLAST+ integrated into Galaxy. Gigascience 4:39 10.1186/s13742-015-0080-7 PMC4557756 26336600

[CR21] Cringoli G, Rinaldi L, Veneziano V, Capelli G, Scala A (2004) The influence of flotation solution, sample dilution and the choice of McMaster slide area (volume) on the reliability of the McMaster technique in estimating the faecal egg counts of gastrointestinal strongyles and *Dicrocoelium dendriticum* in sheep. Vet Parasitol 123(1–2):121–131 10.1016/j.vetpar.2004.05.021 15265576

[CR22] Cringoli G, Rinaldi L, Maurelli M, Utzinger J (2010) FLOTAC: new multivalent techniques for qualitative and quantitative copromicroscopic diagnosis of parasites in animals and humans. Nat Protoc 5(3):503–515 10.1038/nprot.2009.235 20203667

[CR23] Durie PH (1961) Parasitic gastro-enteritis of cattle: distribution and survival of infective strongyle larvae on pasture. Aust J Agric Res 12:1200–1211

[CR24] Flueck WT, Smith-Flueck J (2012) Diseases of red deer introduced to Patagonia and implications for native ungulates. Anim Prod Sci 52:766–773

[CR25] Gasser RB (2006) Molecular tools—advances, opportunities and prospects. Vet Parasitol 136:69–89 10.1016/j.vetpar.2005.12.002 16457951

[CR26] Gasser RB (2025) Advances in molecular tools for parasitic nematodes of animals—from genetic markers to metabarcoding and genomics. Biotechnol Adv 85:108688 10.1016/j.biotechadv.2025.108688 40812747

[CR27] Gasser RB, Chilton NB, Hoste H, Beveridge I (1993) Rapid sequencing of rDNA from single worms and eggs of parasitic helminths. Nucleic Acids Res 21:2525–2526 10.1093/nar/21.10.2525 PMC309567 8506152

[CR28] Gasser RB, Bott NJ, Chilton NB, Hunt P, Beveridge I (2008) Toward practical DNA-based diagnostic methods for parasitic nematodes of livestock. Biotechnol Adv 26:325–334 10.1016/j.biotechadv.2008.03.003 18502076

[CR29] Gray D (2016) Deer populations rising, along with antler size. The Age, Melbourne

[CR30] Irvine RJ, Corbishley H, Pilkington JG, Albon SD (2006) Low-level parasitic worm burdens may reduce body condition in free-ranging red deer (Cervus elaphus). Parasitology 133:465–475 10.1017/S0031182006000606 16817998

[CR31] Kamil M, Nor S, Ahmad M, Mohamad M (2001) Home-range size of captive-bred sambar deer in the wild at Sungkai Wildlife Reserve, Perak. J Wildl Parks 19:19–23

[CR32] McKenzie RA, Green PE, Thornton AM, Chung YS, MacKenzie AR, Cybinski DH, St George TD (1985) Diseases of deer in south-eastern Queensland. Aust Vet J 62:424 10.1111/j.1751-0813.1985.tb14129.x 3833202

[CR33] McLean JL, Avramenko RW, Redman EM, Gilleard JS (2023) High-throughput mitogenomics reveals fine scale population genetic structure and transmission dynamics of gastrointestinal nematodes. Parasit Vectors 16:112

[CR34] McLennan C (2016) Deer hunting: Solving the deer dilemma. The Weekly Times, Melbourne

[CR35] Mes T, Ploeger H, Terlou M, Kooyman F, Van Der Ploeg M, Eysker M (2001) A novel method for the isolation of gastro-intestinal nematode eggs that allows automated analysis of digital images of egg preparations and high throughput screening. Parasitology 123(3):299–314 10.1017/s0031182001008496 11578095

[CR36] Mes TH, Eysker M, Ploeger HW (2007) A simple, robust and semi-automated parasite egg isolation protocol. Nat Protoc 2(3):486–489 10.1038/nprot.2007.56 17406611

[CR37] Moloney P, Turnbull J (2014) Estimates of harvest for deer, duck and quail in Victoria: results from surveys of Victorian game licence holders in 2014. Game Management Authority, Melbourne

[CR38] Morgan ER, Milner-Gulland EJ, Torgerson PR, Medley GF (2004) Ruminating on complexity: macroparasites of wildlife and livestock. Trends Ecol Evol 19:181–188 10.1016/j.tree.2004.01.011 16701252

[CR39] Moriarty A (2004) The liberation, distribution, abundance and management of wild deer in Australia. Wildl Res 31:291–299

[CR40] Morse BW, Nathan P, Nibbelink DA, Osborn KV (2009) Home range and habitat selection of an insular fallow deer population on Little St Simons Island, Georgia. Eur J Wildl Res 55:325–332

[CR41] Odden M, Wegge P (2007) Predicting spacing behavior and mating systems of solitary cervids: a study of hog deer and Indian muntjac. Zoology (Jena) 110(4):261–270 10.1016/j.zool.2007.03.003 17614268

[CR42] Omeragić J, Zuko A (2011) Review of parasitofauna of wild animals in Bosnia and Herzegovina. Vet (Sarajevo) 60:251–257

[CR43] Pybus MJ (1990) Survey of hepatic and pulmonary helminths of wild cervids in Alberta, Canada. J Wildl Dis 26:453–459 10.7589/0090-3558-26.4.453 2250321

[CR44] Ray N, Burgman MA (2006) Subjective uncertainties in habitat suitability maps. Ecol Model 195:172–186

[CR45] Rehbein S, Visser M, Jekel I, Silaghi C (2014) Endoparasites of the fallow deer (*Dama dama*) of the Antheringer Au in Salzburg, Austria. Wien Klin Wochenschr 126:37–41 10.1007/s00508-014-0506-8 24535173

[CR46] Rinaldi L, Coles GC, Maurelli MP, Musella V, Cringoli G (2011) Calibration and diagnostic accuracy of simple flotation, McMaster and FLOTAC for parasite egg counts in sheep. Vet Parasitol 177(3–4):345–352 10.1016/j.vetpar.2010.12.010 21216533

[CR47] Santín-Durán M, Alunda JM, Hoberg EP, de la Fuente C (2008) Age distribution and seasonal dynamics of abomasal helminths in wild red deer from central Spain. J parasitol 94(5):1031–1037 10.1645/GE-1109.1 18576697

[CR48] Shimalov VV, Shimalov VT (2003) Helminth fauna of cervids in Belorussian Polesie. Parasitol Res 89:75–76 10.1007/s00436-002-0700-x 12474047

[CR49] Sleeman DP (1981) Parasites of deer in Ireland. PhD thesis, University College Dublin, Dublin

[CR50] Sommerville E, Condon M (2018) Wild deer approved for human consumption as animal numbers rocket across Victoria. ABC Goulburn Murray

[CR51] Spaggiari J, de Garine-Wichatitsky M (2006) Home range and habitat use of introduced rusa deer in New Caledonia. N Z J Zool 33:175–183

[CR52] Triggs B (1996) Tracks, scats and other traces: a field guide to Australian mammals. Oxford University Press, Melbourne

[CR53] Vengušt G, Bidovec A (2003) Parasites of fallow deer (*Dama dama*) in Slovenia. Helminthologia 40:161–164

