# Supplementary material for: Nemabiome metabarcoding reveals strongyle parasite diversity in wild deer in Victoria, Australia
Source: Parasitol Res. 2026 Jul 8;125(1):118. doi: 10.1007/s00436-026-08722-1 (PMC13630797; doi:10.1007/s00436-026-08722-1)
Supplement: Supplementary file 1 [file 436_2026_8722_MOESM1_ESM.docx]

**Supplementary figure:**

1000

300

50

**+**

**ve**

**L**

**A7**

**A11**

**A14**

**C45**

**C49**

**C51**

**M3**

**M61**

**M15**

**M68**

**M72**

**M75**

**M76**

**M78**

**M69**

**M80**

**M85**

**-**

**ve**

A

1000

300

50

**+**

**ve**

**L**

**M86**

**M91**

**M93**

**M10**

**2**

**M11**

**2**

**N1**

**N5**

**N15**

**N16**

**N19**

**N22**

**N26**

B

**+**

**ve**

**L**

**C1**

**C2**

**C3**

**C1**

**0**

**C1**

**7**

**C1**

**9**

**C2**

**1**

**C2**

**2**

**C2**

**4**

**C2**

**5**

**C26**

**C27**

**C28**

**C29**

**C30**

**C32**

**C33**

**C34**

**C35**

**C36**

**C37**

**C38**

**C39**

**C40**

**-**

**ve**

200

100

300

1000

50

C

**+**

**ve**

**L**

**C41**

**C43**

**C44**

**C46**

**C49**

**C52**

**C53**

**C54**

**C55**

**C56**

**C57**

**C58**

**C59**

**-**

**ve**

100

1000

50

D

**+**

**ve**

**L**

**H1**

**H2**

**H10**

**H13**

**H14**

**H16**

**H18**

**H20**

**H21**

**H24**

**H26**

**H27**

**H28**

**H29**

**H30**

**H31**

**H32**

**H34**

**H35**

**H37**

**-**

**ve**

100

1000

50

300

200

E

**+**

**ve**

**L**

**H3**

**8**

**H4**

**0**

**H4**

**1**

**H4**

**2**

**H4**

**3**

**H4**

**6**

**H4**

**7**

**H4**

**8**

**H4**

**9**

**H5**

**0**

**H5**

**1**

**H5**

**6**

**H5**

**7**

**H5**

**8**

**H5**

**9**

**H6**

**0**

**H6**

**2**

**H6**

**3**

**H6**

**7**

**-**

**ve**

100

1000

300

F

**Supplementary figure 1**: Agarose gel images of NC1/NC2 PCR assay on strongyle egg derived DNA from Sambar deer (A&B) fallow deer (C &D) and Hog deer (E&F) faecal samples Polymerase Chain Reaction (PCR) positive samples. Electrophoresis was done with 2% agarose gel stained with SYBRsafe stain. L represents DNA ladder, with numbers on left of gels indicating fragment size in base pairs. –ve and +ve represents negative and positive controls respectively. Lanes are labelled with deer faecal sample ID, positive samples only shown in the images.
