# Supplementary material for: Nemabiome metabarcoding reveals strongyle parasite diversity in wild deer in Victoria, Australia
Source: Parasitol Res. 2026 Jul 8;125(1):118. doi: 10.1007/s00436-026-08722-1 (PMC13630797; doi:10.1007/s00436-026-08722-1)
Supplement: Supplementary file 2 [file 436_2026_8722_MOESM2_ESM.docx]

**Supplementary Data**

**Supplementary table 1**: Adaptor and primer sequences used to amplify ITS-2 sequences for amplicon NGS sequencing.

| Primer/adaptor | Sequence 5’🡪3’ |
| --- | --- |
| Adp: (F) | TCGTCGGCAGCGTCAGATGTGTATAAGAGACAG |
| Adp: (R) | GTCTCGTGGGCTCGGAGATGTGTATAAGAGACAG |
| NC1Adp | TCGTCGGCAGCGTCAGATGTGTATAAGAGACAGACGTCTGGTTCAGGGTTGTT |
| NC1Adp1N | TCGTCGGCAGCGTCAGATGTGTATAAGAGACAG**N**ACGTCTGGTTCAGGGTTGTT |
| NC1Adp2N | TCGTCGGCAGCGTCAGATGTGTATAAGAGACAG**NN**ACGTCTGGTTCAGGGTTGTT |
| NC1Adp3N | TCGTCGGCAGCGTCAGATGTGTATAAGAGACAG**NNN**ACGTCTGGTTCAGGGTTGTT |
| NC2Adp | GTCTCGTGGGCTCGGAGATGTGTATAAGAGACAGTTAGTTTCTTTTCCTCCGCT |
| NC2Adp1N | GTCTCGTGGGCTCGGAGATGTGTATAAGAGACAG**N**TTAGTTTCTTTTCCTCCGCT |
| NC2Adp2N | GTCTCGTGGGCTCGGAGATGTGTATAAGAGACAG**NN**TTAGTTTCTTTTCCTCCGCT |
| NC2Adp3N | GTCTCGTGGGCTCGGAGATGTGTATAAGAGACAG**NNN**TTAGTTTCTTTTCCTCCGCT |

N representing random nucleotides, used to increase the number and diversity of amplicons generated. Addition of adaptors to primers was as per published studies (Avramenko et al. 2015) and Illumina instructions (Ref:illumina-adapter-sequences-1000000002694-08).

**Supplementary table 2**: Details of reference sequences used in database. Reference sequences were taken from studies which had sequenced parasite DNA from morphologically identified worms. Species highlighted in **bold** indicate possible environmental contaminates, with sequences aligning with these references considered background or contaminates and discarded from analysis.

| **Species** | **Accession** | **Host** | **Country** |
| --- | --- | --- | --- |
|  |  |  |  |
|  |  |  |  |
| *Cooperia punctata* | KY741869 | Cattle | Canada |
| *Cooperia surnabada* | X83560 | Sheep | Australia |
| *Cooperia oncophora* | KC998743.1 | Sheep | New Zealand |
| *Ostertagia gruehneri* | AJ400716 | Reindeer | Svalbard |
| *Ostertagia leptospicularis* | KC998722 | Sheep | New Zealand |
| *Haemonchus contortus* | KF364632 | Cattle | Australia |
| *Haemonchus placei* | KP150469 | Cattle | Canada |
| *Marshallagia marshalli* | AJ400715 | Reindeer | Svalbard |
| *Oesophagostomum radiatum* | KP150518 | Cattle | Canada |
| *Oesophagostomum venulosum (5)* | KC998755 | Sheep | New Zealand |
| *Ostertagia osteragi* | KP150491 | Cattle | Canada |
| *Spiculopteragia asymmetrica* | AJ251125 | Red Deer | Australia |
| *Spiculopteragia spiculoptera* | DQ354310 | Roe Deer | France |
| *Teladorsagia circumcincta* | X86026 | Sheep |  |
| *Trichostrongylus axei* | Y15875 | Sheep | Victoria, Australia |
| *Trichostrongylus capricola* | JF276022 | Sheep | Iran |
| *Trichostrongylus colubriformis* | X78063 | Sheep | Aust |
| ***Trichostrongylus retortaeformis*** | X78066 | Rabbit | Victoria, Australia |
| *Trichostrongylus vitrinus* | X78064 | Sheep | Victoria, Australia |
| *Ostertagia antipini* | JQ925869 | Moose | Russia |
| *Spiculopteragia houdemeri* | AB682702 | Sika Deer |  |
| *Skrjabinagia sp* | AB367797 | Japanese Serow | Japan |
| ***Cloacina hydriformis*** | Y10796 |  |  |
| ***Rugopharynx macropodis*** | LN906963 |  |  |
| ***Rugopharynx rosemariae*** | Y10792 |  |  |
| ***Strongylida environmental sample*** | JX155302 |  |  |
| ***Heterodera betae*** | LC208691 |  |  |
| ***Heterodera ciceri*** | AY045758 |  |  |
| ***Heterodera glycines*** | AF216579 |  |  |
| ***Heterodera schachtii*** | EF611123.1 |  |  |
| ***Heterodera trifolii*** | AF498388.1 |  |  |
| ***Macropostrongyloides baylisi*** | Y10800 |  |  |
| *H Placei* | KF364627 | Cattle |  |
| *Trichostrongylus axei* | X78065 | Sheep |  |

**Supplementary table 3:** Number of reads per individual sample following quality filtering.

|  | **Hog** | **Fallow** | **Sambar** |  |
| --- | --- | --- | --- | --- |
|  | 150920 | 65066 | 72653 |  |
|  | 58976 | 56609 | 57049 |  |
|  | 114245 | 25326 | 48880 |  |
|  | 308853 | 30376 | 3528 |  |
|  | 93316 | 17145 | 36377 |  |
|  | 144295 | 53924 | 76386 |  |
|  | 57277 | 91705 | 3847 |  |
|  | 70234 | 184830 | 3507 |  |
|  | 180164 | 118387 | 4125 |  |
|  | 154000 | 42586 | 32430 |  |
|  | 45554 | 104927 | 40599 |  |
|  | 30938 | 66969 | 19482 |  |
|  | 59048 | 56025 | 29456 |  |
|  | 84145 | 85233 | 108522 |  |
|  | 423499 | 46775 | 266717 |  |
|  | 59993 | 67127 | 21322 |  |
|  | 192084 | 32599 | 31541 |  |
|  | 71925 | 61623 | 62336 |  |
|  | 64564 | 96206 | 15895 |  |
|  | 88306 | 115734 | 55443 |  |
|  | 99671 | 36198 | 64443 |  |
|  | 38509 | 91017 | 13528 |  |
|  | 83314 | 55981 | 40670 |  |
|  | 155053 | 32967 | 15166 |  |
|  | 49137 | 49856 | 24318 |  |
|  | 849543 | 11549 | 27939 |  |
|  | 22273 | 67712 | 23745 |  |
|  | 91220 | 53459 | 62430 |  |
|  | 364376 | 64040 | 40860 |  |
|  | 60683 | 69423 |  |  |
|  | 31731 | 60980 |  |  |
|  | 88894 | 93783 |  |  |
|  | 28612 | 43257 |  |  |
|  | 81821 | 60271 |  |  |
|  | 6583 | 25750 |  |  |
|  | 72602 | 62940 |  |  |
|  | 76356 | 65145 |  |  |
|  | 578080 |  |  | **Total** |
| **Total reads** | 5230794 | 2363500 | 1303194 | 8897488 |
| **Average** | 137652.5 | 63878.38 | 44937.72 | 85552.77 |
| Standard Deviation | **167066.85** | **33088.23** | 49577.20 | 112746.84 |
|  | **Toora** | **Tonimbuk** | **Neerim** |  |
| **Deer** | 111213 | 16679 | 8456 |  |
| **Cattle** | 216044 | 100919 | 83899 |  |
